# Ceramic-Based 4D Components: Additive Manufacturing (AM) of Ceramic-Based Functionally Graded Materials (FGM) by Thermoplastic 3D Printing (T3DP)

**DOI:** 10.3390/ma10121368

**Published:** 2017-11-28

**Authors:** Uwe Scheithauer, Steven Weingarten, Robert Johne, Eric Schwarzer, Johannes Abel, Hans-Jürgen Richter, Tassilo Moritz, Alexander Michaelis

**Affiliations:** 1 Processes and Components, Fraunhofer Institute for Ceramic Technologies and Systems (IKTS), 01277 Dresden, Germany; steven.weingarten@ikts.fraunhofer.de (S.W.); eric.schwarzer@ikts.fraunhofer.de (E.S.); johannes.abel@ikts.fraunhofer.de (J.A.); hans-juergen.richter@ikts.fraunhofer.de (H.-J.R.); tassilo.moritz@ikts.fraunhofer.de (T.M.); alexander.michaelis@ikts.fraunhofer.de (A.M.); 2 Fraunhofer Singapore, Singapore 639798, Singapore; robert.johne@fraunhofer.sg

**Keywords:** additive manufacturing (AM), functionally graded materials (FGM), thermoplastic 3D printing (T3DP), ceramics, ceramic-based 4D components, zirconia, graded microstructure

## Abstract

In our study, we investigated the additive manufacturing (AM) of ceramic-based functionally graded materials (FGM) by the direct AM technology thermoplastic 3D printing (T3DP). Zirconia components with varying microstructures were additively manufactured by using thermoplastic suspensions with different contents of pore-forming agents (PFA), which were co-sintered defect-free. Different materials were investigated concerning their suitability as PFA for the T3DP process. Diverse zirconia-based suspensions were prepared and used for the AM of single- and multi-material test components. All of the samples were sintered defect-free, and in the end, we could realize a brick wall-like component consisting of dense (<1% porosity) and porous (approx. 5% porosity) zirconia areas to combine different properties in one component. T3DP opens the door to the AM of further ceramic-based 4D components, such as multi-color, multi-material, or especially, multi-functional components.

## 1. Introduction

The term “4D printing” was initially used by Tibbits and Sheil [1] and describes a technology that was developed through a collaboration between The Self-Assembly Lab, Stratasys, and Autodesk to manufacture customizable smart materials. By using an additive manufacturing (AM) device, polymer multi-material components with the added capability of shape transformation from one state to another can be realized. This offers the possibility to include functionalities such as actuation, sensing, and material logic directly into the components [2]. Additively manufactured multi-material components can transform from any 1D strand or 2D surface into a 3D shape, or morph from one 3D shape into another. Using only water, heat, light, or another simple energy input, this technique offers adaptability and dynamic response for structures and systems of all sizes [2]. Khoo et al. conducted an overview of actual works on “4D printing” based on polymer and metallic materials [3]. Khare et al. presented different concepts and technologies to combine different materials on the atomic scale to realize components that can change their shape or operate as an actuator [4].

For ceramic components in particular, it is very challenging to realize such properties. The reason for this challenge is that a thermal treatment is necessary after the AM process to generate the ceramic properties. In order to obtain a multi-material composite, it is fundamental to successfully co-process and co-sinter the paired powders in the composite material. Since the sintering of the components is performed at the same temperature and atmosphere, it is a prerequisite for all of the materials to have a comparable sintering temperature and behavior (starting temperature of sintering, shrinkage behavior). To avoid critical mechanical stress during cooling, it is also important that the coefficient of thermal expansion of all the materials is approximately equal [5,6].

Nevertheless, it is possible to realize ceramic components with a variety of properties by realizing a graded microstructure or material gradients. These composites are called functionally graded materials (FGM) [7]. To achieve unprecedented properties of ceramic components, we combine the benefits of AM with the benefits of FGM to create “ceramic-based 4D materials”, which are manufactured additively and combine different properties and/or materials in one component after a co-sintering step.

**Figure 1:**
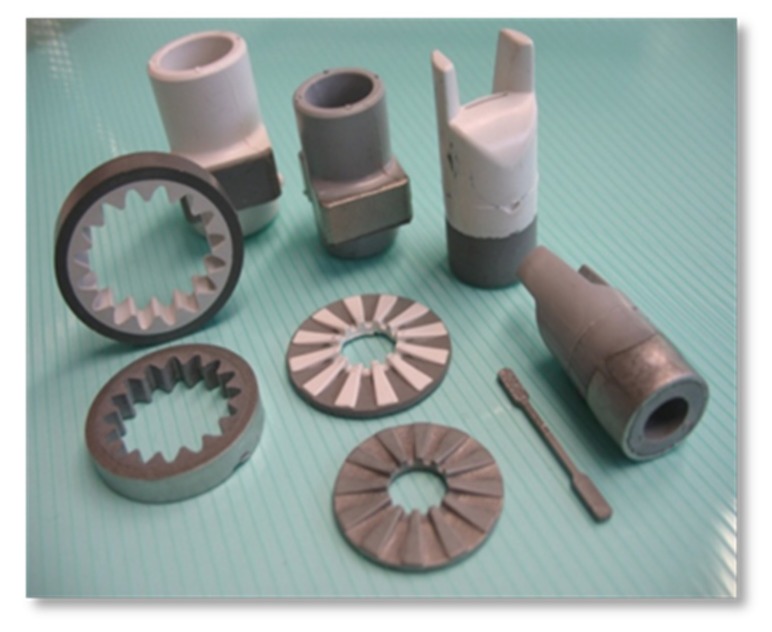
Metal–ceramic composites manufactured by two-component ceramic injection molding (2C-CIM) (manufactured by group “Shaping” at FhG-IKTS).

**Figure 2:**
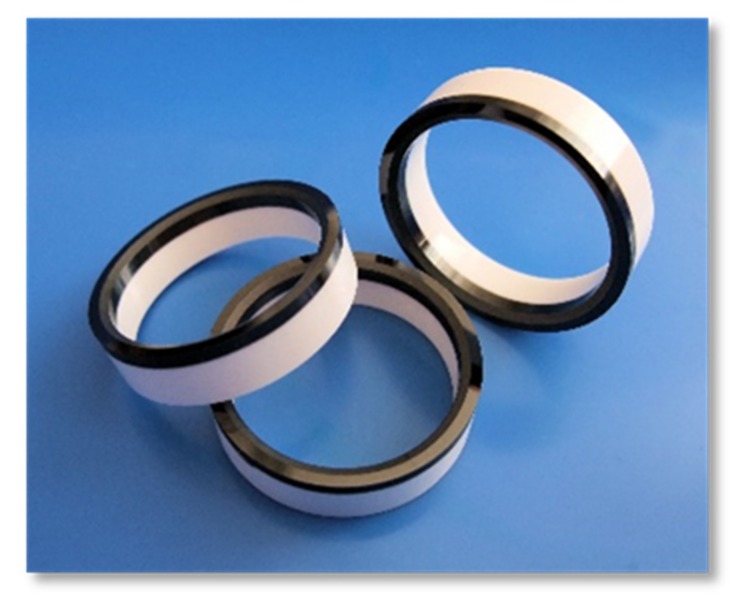
Two-colored zirconia components manufactured by 2C-CIM (manufactured by group “Shaping”, FhG-IKTS).

In a FGM, the properties change gradually with position [7], which generates new fields of application. The combination of two or more materials in graded microstructures such as ceramic–metal composites results in innovative, multi-functional property combinations, such as hard and ductile, electrically or thermally conductive and insulating, magnetic and nonmagnetic [8]. Such materials could be used to create conceivable applications in a variety of industrial and medical fields, such as for example, cutting tools, wear-resistant components, energy and fuel cell components, and bipolar surgical tools [9,10,11,12,13,14]. Figure 1 shows the metal–ceramic components for different applications manufactured at our group “Shaping” of the Fraunhofer IKTS (Institute for Ceramic Technologies and Systems) by two-component ceramic injection molding (2C-CIM) [14]. Furthermore, the manufacturing of two-color components is possible with 2C-CIM, which was demonstrated by Fraunhofer IKTS (FhG-IKTS) as well. (Figure 2).

**Figure 3:**
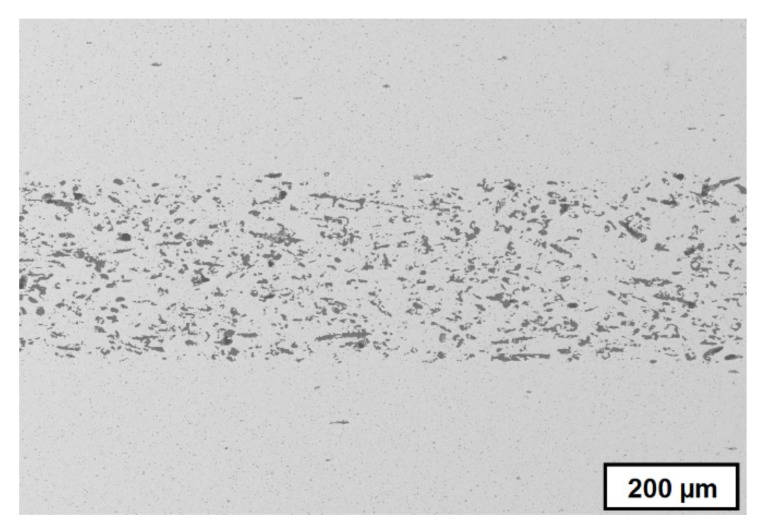
Cross-section of sintered MgO–ZrO_2_ component with graded microstructure (manufactured by group “Shaping”, FhG-IKTS).

**Figure 4:**
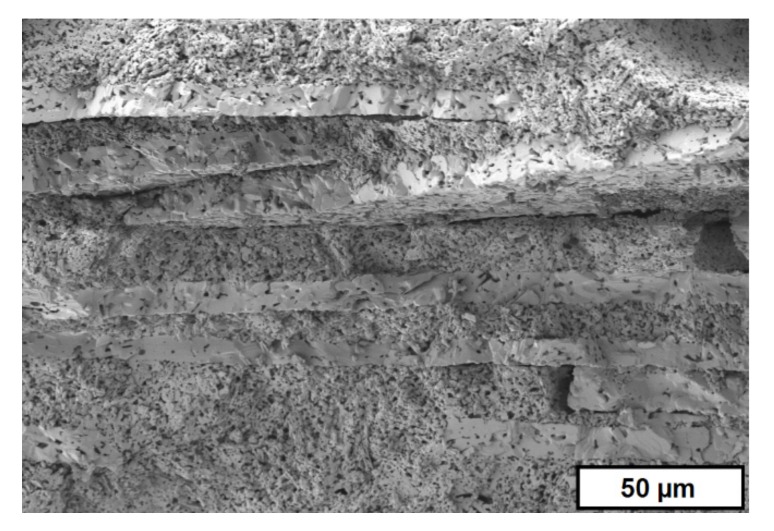
Crack surface of CaAl–Al_2_O_3_ component after a bending test (manufactured by group “Shaping”, FhG-IKTS).

Components with different porosities combine different properties in the gradient structure regarding thermal conductivity, heat capacity, density, mechanical strength, and elastic modulus, which results in improved thermal shock properties [15]. In the case of open porosity, it is possible to generate selective filters that are able to filtrate by tailored pore sizes in a complex or small single component. Figure 3 shows the SEM image of a cross-section of a sintered MgO–ZrO_2_ component with graded porosity, and Figure 4 shows the crack surface after a crack deflection during a bending test, which occurred due to the graded microstructure and varied Young’s modulus or rather stress distribution [8]. Both samples were manufactured at Fraunhofer IKTS.

**Figure 5:**
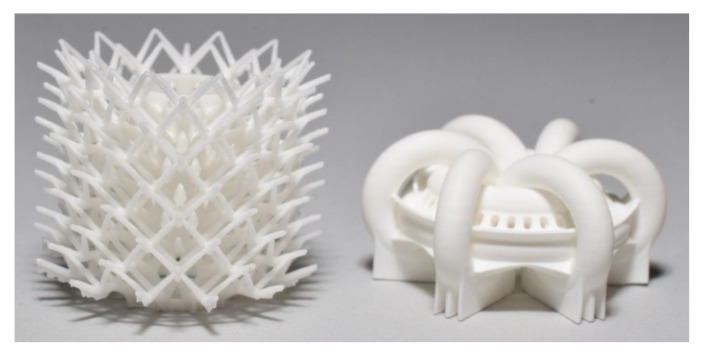
Alumina heat exchangers additively manufactured by lithography-based ceramic manufacturing (LCM) at Fraunhofer IKTS.

**Figure 6:**
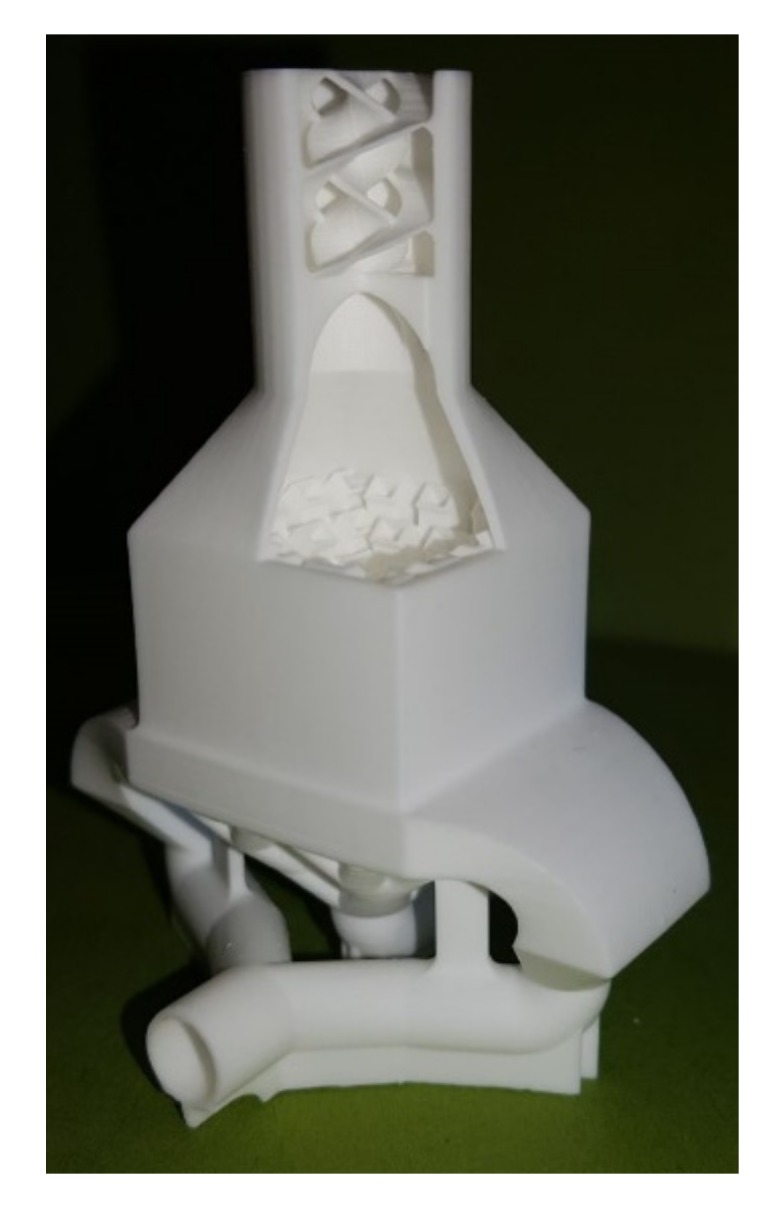
Alumina static mixer additively manufactured by lithography-based ceramic manufacturing (LCM) at Fraunhofer IKTS.

AM technologies make it possible to produce components with extremely complex geometry that cannot be attained by any other shaping technique without the use of tools, because it is “the general term for those technologies that based on a geometrical representation creates physical objects by successive addition of material” [16]. For processing ceramic materials, the technical application of AM technologies has been limited so far. However, ceramics have been studied in AM processes ab initio, with the development of different AM technologies over the last 25 years [17,18]. All of the established AM technologies have also been tested for ceramic materials [19,20,21,22,23,24,25,26,27,28,29,30,31,32,33,34,35,36,37,38,39,40,41,42,43,44,45]. AM technologies can be classified according to the state of the material that is used—powder materials, liquid materials and solid materials [46,47]—or according to the kind of material deposition and solidification [48]. Lithography-based ceramic manufacturing (LCM) enables the AM of dense alumina (Figure 5 and Figure 6) and zirconia components with extraordinary complex geometries [24,25,26,49,50].

Several technologies are known for the production of FGM. There are very good overviews by Kieback et al. [7], Naebe et al. [51], and Moritz et al. [5]. Conventional shaping technologies can be used to produce FGM, such as powder pressing [52], slip casting [53,54], powder injection molding [55,56], tape casting [8,57,58,59]. A combination of conventional shaping technologies can also be used to produce FGM. One example is inmold labeling, which is a combination of tape casting and injection molding [60,61].

The first studies on the AM of ceramic-based FGM components were published about 20 years ago [62,63,64,65]. The multiphase jet solidification (MJS) technology, which is based on thermoplastic binder systems filled with high contents of ceramic particles, is nearly similar to the fused filament fabrication (FFF)/fused deposition modeling (FDM^®^) technology. A hot powder–binder mixture (feedstock) with suitable flow properties was deposited with an extrusion jet that moved in two dimensions. During cooling, the mixture solidified, and a free-standing body was formed. By using a two-piston construction combined with a small static mixing chamber, the powder composition was varied from layer to layer, and 3D graded SiC–TiC components could be formed [62,63].

Zhang et al. used laminated object manufacturing (LOM) to manufacture TiC–Ni FGMs. Green tapes with different compositions were produced, cut by a laser, stacked, and laminated [64].

Cesarano, J. III et al. already demonstrated in 1998 that a multi-material application is feasible with robocasting. For example, in addition to alumina components with different geometries (dense or porous), alumina/TiCuSil composite could also be realized [65].

Polsakiewicz and Kollenberg presented the AM of alumina-based multi-material components by binder jetting [45,66]. Ceramic or metal powders were dispersed in inks and selectively deposited in a ceramic powder bed.

**Figure 7:**
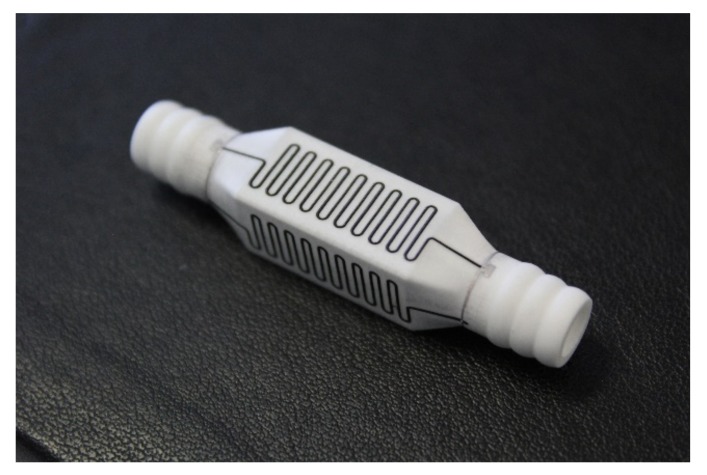
Post-functionalized additively manufactured alumina component (Fraunhofer IKTS).

Another approach is the functionalization of additively manufactured and sintered ceramic components subsequent to their generation. Figure 7 shows an alumina component manufactured by lithography-based ceramic manufacturing (LCM) that was sintered at 1600 °C and subsequently functionalized with electrically conductive heater structures by aerosol printing [5].

Direct AM technologies are more suitable for the AM of multi-material components than indirect AM technologies because of the selective deposition of the used material. The latter are based on the selective solidification of material deposited all over the entire layer. Therefore, the areas that have not been solidified are occupied by non-solidified material, which have to be removed before a second material can be deposited in these areas. Using direct AM technologies, a second material and further ones can be deposited directly beside the already deposited and solidified first material.

In our study, we investigated the AM of ceramic-based FGM by thermoplastic 3D printing (T3DP), which is a direct AM technology. Zirconia components with varying microstructures were additively manufactured by using thermoplastic suspensions with different contents of pore-forming agents (PFA), and were co-sintered defect-free.

## 2. Experimental

### 2.1. Thermoplastic 3D-Printing

T3DP is based on the selective deposition of particle-filled thermoplastic suspensions not over the entire surface, but instead only at the required spots [67]. Different suspensions can be deposited beside each other in each single layer, and bulk material as well as property gradients can be realized within the additively manufactured green components [67,68,69]. The solidification takes place almost independently of the physical properties of the used powders, so even hard metal components can be manufactured [70].

**Figure 8:**
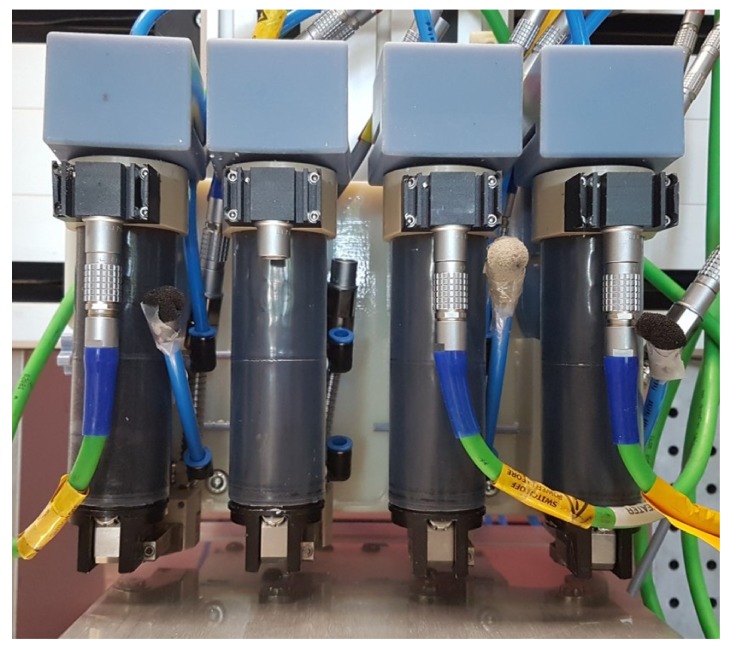
Thermoplastic 3D printing (T3DP) device with four different micro dispensing units.

A special device was developed in Vermes, Germany that combines several supply containers and mechanical and electronic micro dispensing units (Figure 8). Hence, it could be used for the spatially resolved deposition of different materials, including supporting structures, in the same component. The micro dispensing units work with a nozzle orifice diameter of 160 μm. A piezo-driven hard metal piston moves up and down in the nozzle chamber to jet single droplets instead of filaments, which are deposited with extruder-like systems [71,72]. In our study, we used a maximum frequency of 100 Hz, but a frequency up to 3000 Hz is possible with the used system.

### 2.2. Used Materials

For the ceramic material, yttria-stabilized zirconia powders TZ-3Y-E by Tosoh Corporation of Japan (Tokyo, Japan) were used.

Three different materials were investigated concerning their behavior as PFAs. The main focus here was on the need for the PFA particles’ diameter to be much smaller than the nozzle orifice diameter of the micro dispensing system (160 μm); in particular, a particle diameter of 25 μm has proven applicable in previous experiments. In addition, the melting temperature of the PFA material should be higher than the melting temperature of the binder systems’ components to avoid undesired pore formation by the heating during processing, as well as inhomogeneities during the printing. ARBOCEL UFC100 of the company J. Rettenmaier Söhne GmbH + Co KG (Rosenberg, Germany) is a cellulose powder based on cellulose fibers with an average particle size of 6–12 μm. The decomposition temperature of this material is about 200 °C, and corresponds to the decomposition temperature of a second polysaccharide (PS) powder (starch) with a rounder shape and a maximum diameter below 25 μm. CERETAN MA 7008 (Münzing Chemie GmbH, Abstatt, Germany) is an amide of a carboxylic acid with a decomposition temperature of about 150 °C and a d_99_ of 8 μm. It was selected to investigate its suitability as a PFA in spite of the significantly lower decomposition temperature.

### 2.3. Feedstock Preparation

The different zirconia suspensions were prepared by dispersing the powder together with the PFA in a thermoplastic binder system based on a mixture of molten paraffin and beeswax. The dispersion and homogenization took place in a heatable dissolver (Dissolver DISPERMAT CA 20-C, VMA-Getzmann GmbH, Reichshof, Germany) by stirring for 4 h. Suspensions with 36 vol.-% and 40 vol.-% were prepared.

The content of the PFA was varied for each material. Table 1 summarizes the composition of the different thermoplastic suspensions.

### 2.4. Preparation of Single- and Multi-Material Test Components

The test samples were additively manufactured by using micro dispensing systems from Vermes, Germany. Four of them were mounted on our xyz-laboratory rig, which can operate alternately. In case of multi-material printing two dispensing units were used simultaneously.

The dispensing parameters had to be adjusted for each suspension due to their different rheological behaviors. Based on experience from previous experiments, the dispensing parameters were chosen to obtain a reproducible favorable droplet shape (small diameter, roundness) and avoid different adverse events such as occurring satellite droplets, adherent material on the nozzle orifice, trapped air, and incomplete fusion between adjacent droplets [72]. The deposition of the droplets had a frequency of up to 100 Hz, and the axes moved with a maximal velocity of 50 mm/s.

The green samples were debinded in a powder bed at a very low heating rate, in a first step under air atmosphere of up to 270 °C (heating rate 4 K/h), and then after removing the powder in a second step under air atmosphere of up to 900 °C (12 K/h). Afterwards, the components were sintered under air atmosphere at 1350 °C (3 K/min) for 2 h.

### 2.5. Characterization Methods

The particle size distribution of the utilized powders and PFA were measured by a laser diffraction method (Mastersizer 2000, Malvern Instruments Ltd., Malvern, UK). Electron scanning microscopy images have been used to characterize the shape of the particles.

To characterize the rheological behavior of the zirconia suspensions, a rheometer (Modular Compact Rheometer MCR 302; Anton Paar, Graz, Austria) that was adjustable between −25 to 200 °C with a plate/plate measuring system was used. The flow behavior was analyzed with an increasing shear rate (0–10,000 s^−1^) and at varying temperatures between 85–100 °C. The torque was measured and the viscosity calculated.

Field Emission Scanning Electron Microscopy (FESEM) images of the sintered samples have been utilized to evaluate the samples’ density and porosity. The FESEM images were converted into binary images by means of an open source software called ImageJ (1.47v, open source software, National Institutes of Health, USA), i.e., all of the pores were converted into black pixels, and the ceramic particles were converted into white pixels. In order to calculate the samples’ porosity, the software compared the number of the black and white pixels in a converted image of a samples’ cross-sections.

Additionally, the density was measured by Archimedes’ principle.

## 3. Results and Discussion

### 3.1. FESEM Studies of Used Materials

**Figure 9:**
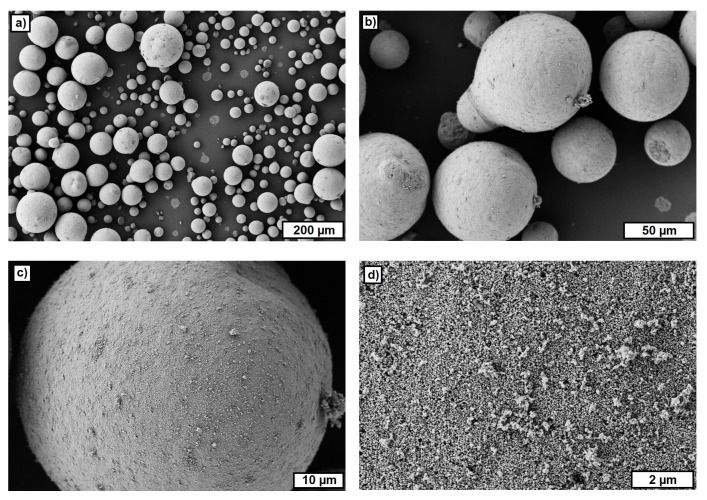
FESEM images of untreated zirconia powder. (**a**) ZrO_2_-granulates, overview; (**b**) ZrO_2_-granulates, detail; (**c**) single ZrO_2_-granulate; (**d**) surface of ZrO_2_-granulate.

The measured average particle size (d_50_) of the zirconia powder was 0.37 μm by about one order of magnitude larger than the average particle size, as stated by Tosoh (d_50_ = 0.04 μm). FESEM images showed that the untreated powder consisted of very large granulates (diameter up to 100 μm) that were crushed during the ultrasonic treatment before the laser diffraction (Figure 9). Still, the applied treatment was insufficient regarding the deagglomeration of all the particles. However, during the feedstock preparation, very high shear forces were realized, which should deagglomerate all particles.

**Figure 10:**
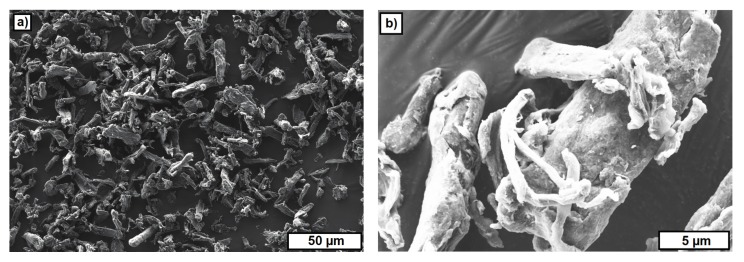
FESEM images of ARBOCEL UFC100. (**a**) Overview; (**b**) detail.

**Figure 11:**
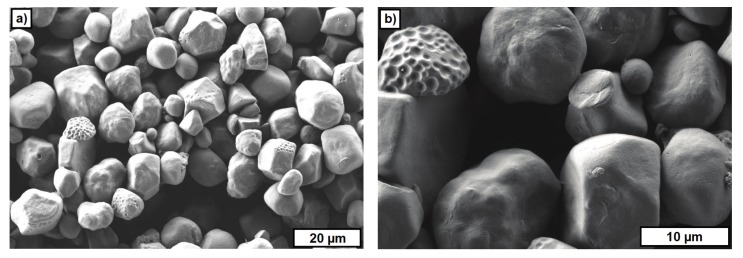
FESEM images of polysaccharide powder. (**a**) Overview; (**b**) detail.

Figure 10 and Figure 11 show FESEM images of two materials used as a PFA. ARBOCEL UFC100 bases on cellulose fibers and the particles have a fiber fragments-like shape (Figure 10). The particles of the PS powder have a polyhedral-like shape (Figure 11). The particle size investigated on FESEM images varied between 5–20 μm.

The FESEM images of the used PFA materials provide an explanation for the content of the PFA, which could be realized in the different suspensions. A significantly higher content of PFA (up to 10 vol.-%) could be realized with the starch powder (polysaccharide). For the other two materials, the viscosity significantly increased for contents of more than 2 vol.-%. This probably results from the higher and rough surface of CERETAN MA 7008 and UFC 100 compared with the smooth surface of the starch powder.

### 3.2. Rheological Behavior of the Thermoplastic Suspensions

**Figure 12:**
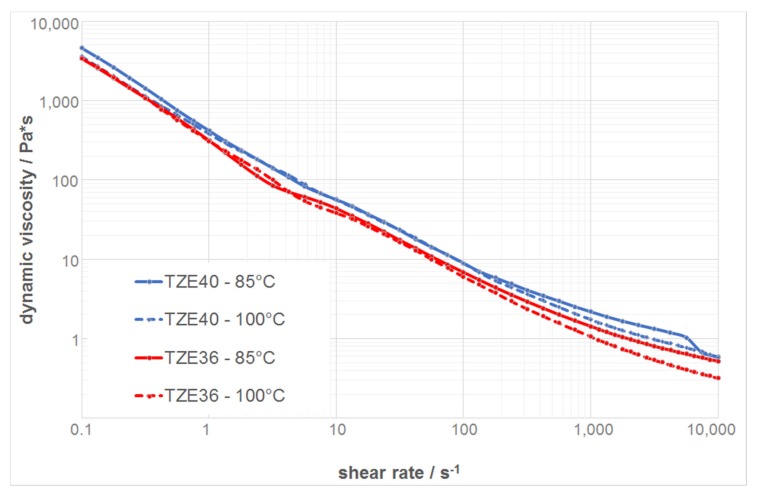
Rheological behavior of zirconia suspensions without any PFA.

The results of the rheological measurements of the two zirconia suspensions without any PFA are summarized in Figure 12. The dynamic viscosity is presented as a function of the shear rate in a double logarithmic plot, with a shear rate range between 0.1 and 10,000 s^−1^, where this range corresponds to the shear rate values at the nozzle for the dispensing parameters used. All of the suspensions show a shear thinning behavior at both temperatures. The dynamic viscosity increases with increasing solid content and decreasing temperature.

**Figure 13:**
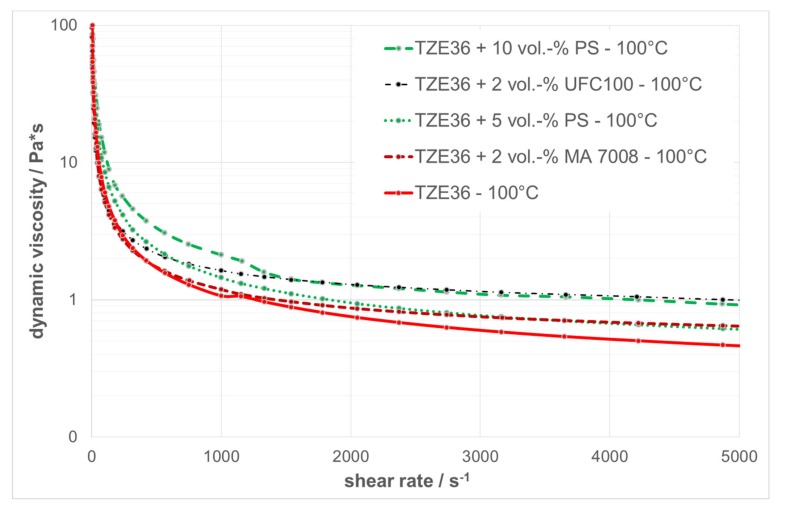
Rheological behavior of zirconia suspensions with and without PFA at 100 °C.

Figure 13 shows the dynamic viscosities as a function of the shear rate at a temperature of 100 °C for the different suspensions with and without PFA. The suspension without any PFA has the lowest viscosity, and by adding PFA, the viscosity increases due to the lower solid content compared with those loaded with PFA. However, all of the suspensions show a shear thinning behavior with a dynamic viscosity of 1 Pa·s or lower at a shear rate of 5000 s^−1^. Moreover, the course of the curves resembles those of previous experiments with printable feedstocks, which therefore can be utilized for T3DP [68]. Table 2 summarizes the measured viscosities of the suspensions at different shear rates and temperatures.

### 3.3. Single-Material Components

**Figure 14:**
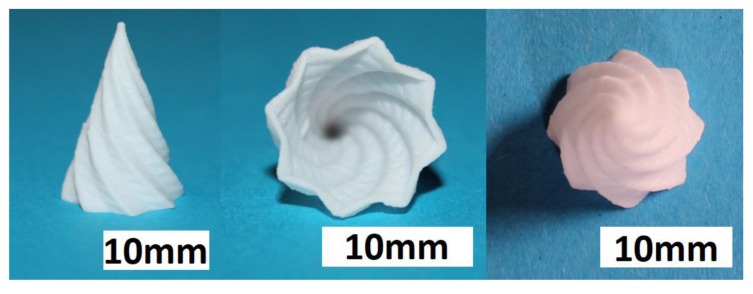
Sintered zirconia test component additively manufactured by thermoplastic 3D printing (T3DP) with 36 vol.-% zirconia particles in the suspension and without PFA; three different views.

After a screening of the deposition parameters, it was possible to manufacture test components for each suspension. Figure 14 shows three views of a complex structure additively manufactured by T3DP with the zirconia suspension without a PFA. The shown test components were realized without a prerequisite utilization of any support structure; respectively, support material. After sintering, the wall thickness of this structure was less than 0.8 mm. The manufacturing time for the green component was approximately 30 min.

**Figure 15:**
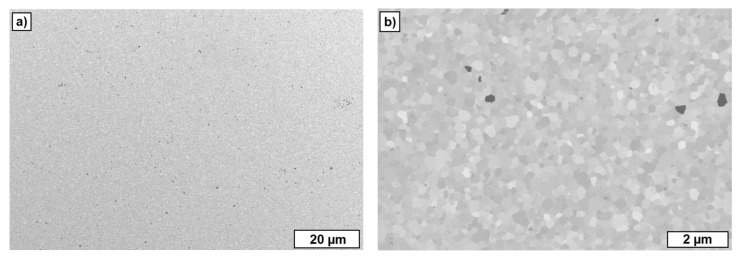
FESEM images of sintered zirconia test components additively manufactured by T3DP with 36 vol.-% zirconia particles and without PFA in the suspension. (**a**) Overview; (**b**) detail.

The FESEM images of the cross-section of sintered zirconia test components additively manufactured by T3DP with 36 vol.-% zirconia particles and without any PFA shown in Figure 15. The microstructure shows a homogeneous structure with a low content of porosity. By utilizing ImageJ and five different FESEM images, an average porosity of 0.11 ± 0.04% was calculated. With Archimedes’ principle, an average outer density of 5.90 ± 0.04 g/cm^3^ and an average inner density of 5.93 ± 0.05 g/cm^3^ was investigated for 14 test samples.

**Figure 16:**
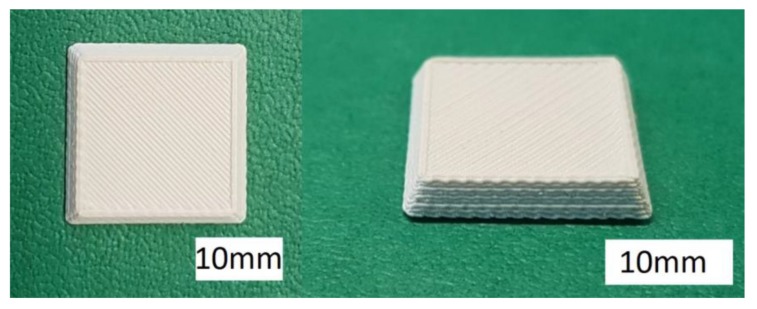
Green zirconia test components with 5 vol.-% polysaccharide (PS) as a PFA; (**left**) top view, (**right**) angled view.

In addition, test samples of the suspensions with PFA were manufactured. Figure 16 shows the top and an angled view of a green sample including 5 vol.-% PS as PFA, for instance.

**Figure 17:**
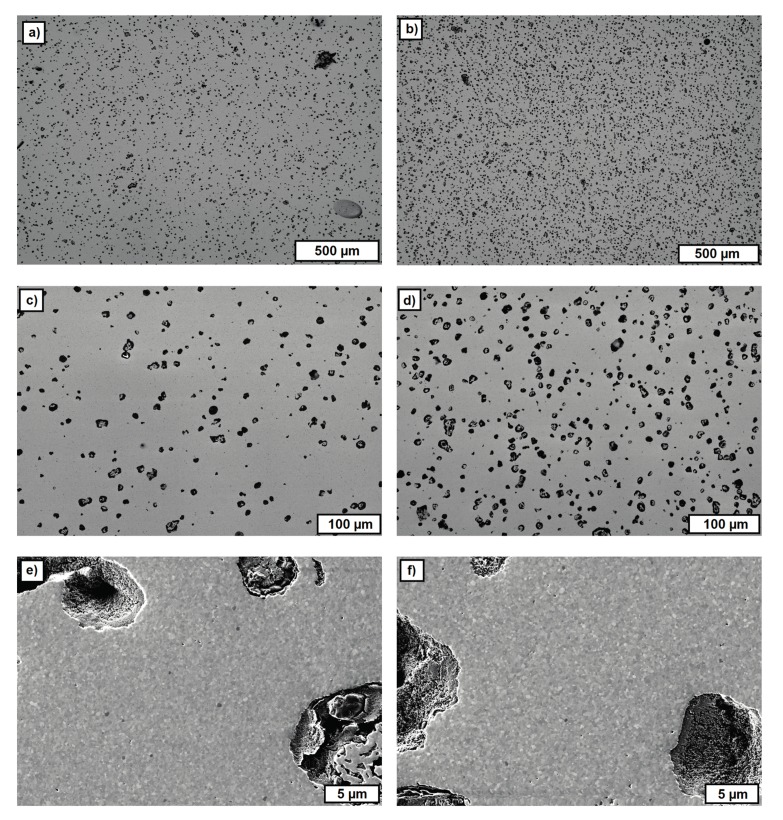
FESEM images of cross-sections of sintered single-material zirconia test samples with 5 vol.-% PS (**left**; (**a**,**c**,**e**)) and 10 vol.-% PS (**right**; (**b**,**d**,**f**)) as PFA in the suspension, with increasing magnifications from top to bottom.

Figure 17 summarizes the FESEM images from regions of the inner core of the sintered single-material test components additively manufactured by T3DP with the suspensions that included 5 vol.-% PS (left) and 10 vol.-% PS (right) as PFA, taken at different magnifications. The images show a homogeneous distribution of the single pores, with a nearly round shape resulting from the polyhedron-like shape of the PS powder as PFA. The average porosities calculated with ImageJ on five images (200 × magnification, 2nd line in Figure 17) each are 5.02 ± 0.43% and 10.72 ± 0.87%, respectively. These correspond almost exactly to the content of added PFA in the suspensions (5 vol.-% and 10 vol.-%).

**Figure 18:**
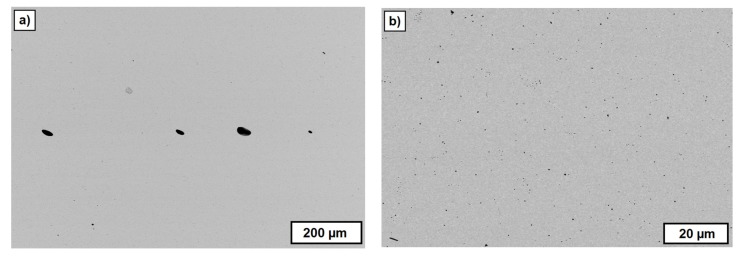
FESEM images of cross-sections of a test component with CERETAN MA 7008 as the PFA. (**a**) Overview; (**b**) detail.

However, not every one of the used materials operated as a PFA. Figure 18 shows the FESEM images of sintered zirconia components additively manufactured by T3DP, and a suspension with 2 vol.-% of CERETAN MA 7008 as PFA. After sintering, only a few pores can be detected, which are located in the image in an almost horizontal row. These are most likely resulting from trapped air bubbles between two layers during the manufacturing process.

The decomposition temperature of CERETAN MA 7008 (about 150 °C) seems to be too low to be utilized as a PFA for T3DP. The material is removed during the first debinding step and probably due to a still lasting thermoplastic behavior of the suspension at this temperature, the rearrangement processes can occur driven by capillary forces. Subsequently, the remaining pores are eliminated during the final sintering step. This topic has to be investigated in further studies.

### 3.4. Ceramic-Based 4D Components

**Figure 19:**
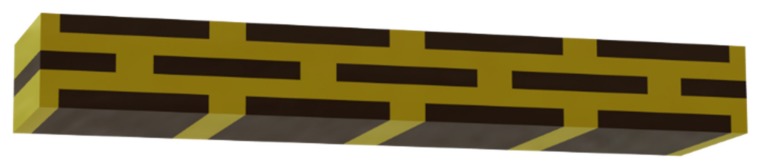
CAD drawing of a 4D component: brick wall-like test component.

**Figure 20:**
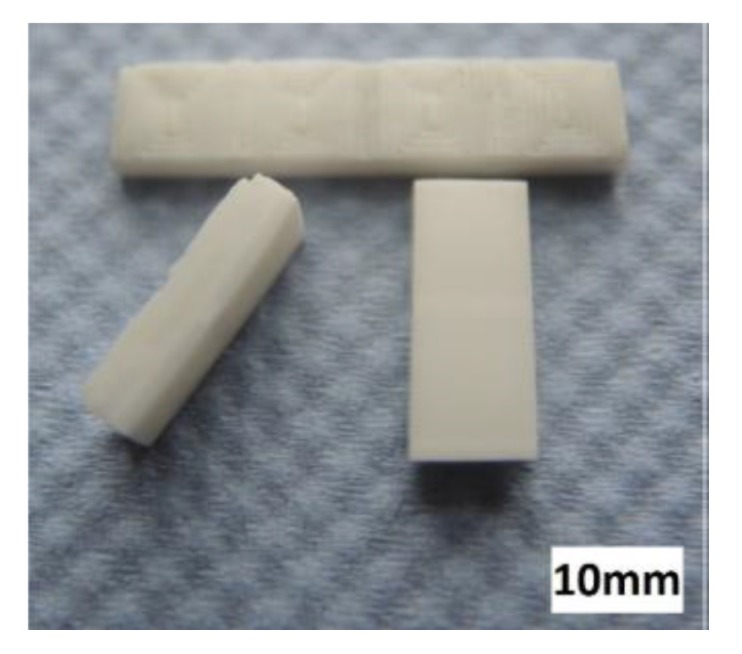
Sintered zirconia 4D components.

To demonstrate the additive manufacturing of zirconia-based 4D-components, we manufactured components with a brick wall design. Figure 19 shows a Computer Aided Design (CAD) drawing of the design. The brighter areas were manufactured with 5 vol.-% PS as the PFA, and the dark areas were manufactured with the pure zirconia suspension (36 vol.-% solid load, no PFA). As previously discussed, CERETAN MA 7008 was not suitable as PFA, and for ARBOCEL UFC100, only 2 vol.-% could be added to the suspension without significantly increasing the viscosity. The suspension with 5 vol.-% PS as PFA already formed a clearly detectable number of pores; therefore, it was considered sufficient for the mere demonstration of producing a 4D component. The resulting defect-free sintered components are shown in Figure 20.

**Figure 21:**
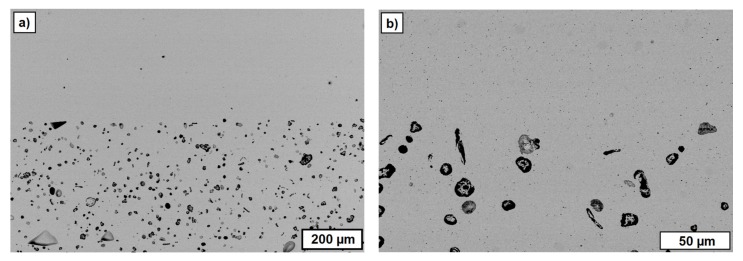
FESEM images of cross-sections of sintered zirconia 4D components; the interface between dense and porous areas; (**a**) overview; (**b**) detail.

FESEM images of cross-sections of these 4D components show clearly distinguishable areas at the interface between the two former suspensions (Figure 21). Thus, regardless of the drop-bound deposition of the material, the arrangement of the different microstructure can be realized very precisely.

**Figure 22:**
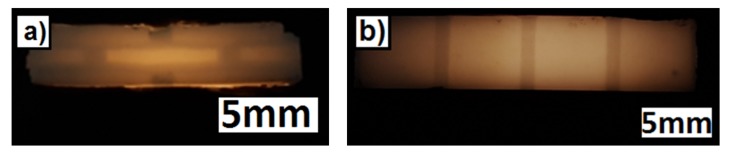
Sintered zirconia 4D component in front of a light spot with two different microstructures (darker areas: porous; brighter areas: dense); (**a**) side view (section); (**b**) top view.

**Figure 23:**
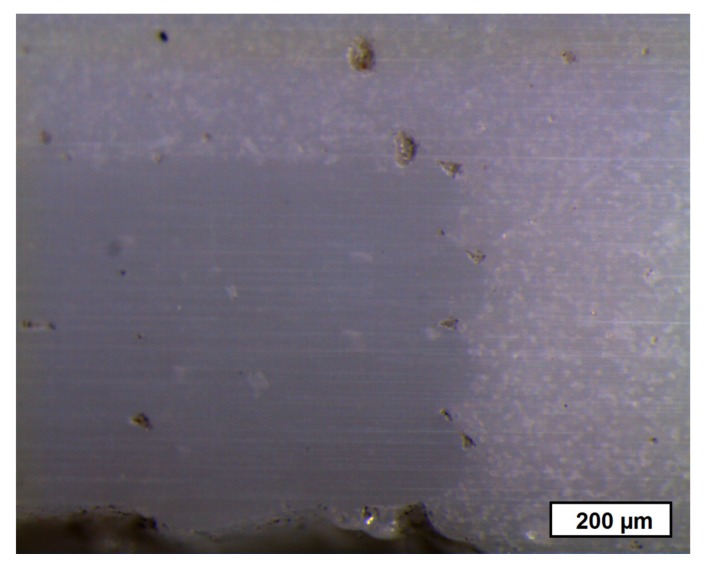
Light microscope image of the interface between the dense (lower left) and porous (top and right) areas within the component.

To illustrate the difference in porosity, we placed the zirconia-based 4D components in front of a light spot (Figure 22). While the porous areas shine darker because of the light deflection and reflection, the denser areas show the opposite behavior by appearing more translucent. Figure 23 shows a light microscope image of the interface between the dense (lower left) and porous area (top and right) within the component.

## 4. Conclusions

In our study, we showed that it is possible to combine AM and FGM to create zirconia-based 4D components. We used the T3DP technology as a directly working AM technology to selectively deposit two different materials beside each other. This offers the possibility of combining suspensions with different contents of a PFA to realize components with dense and porous areas inside.

Different materials were investigated concerning their suitability as PFA for the T3DP process. Different zirconia-based suspensions were prepared and used for the AM of single- and multi-material test components. All of the samples were sintered defect-free, and in the end, we could realize a brick wall-like component consisting of dense (<1% porosity) and porous (approx. 5% porosity) zirconia areas to combine different properties in one component.

T3DP opens the door to the AM of further ceramic-based 4D components such as multi-color or multi-material components, which will be presented in further papers.

## Figures and Tables

**Table 1:** Composition of used thermoplastic suspensions. PFA: pore-forming agent.

**Zirconia Content/vol.-%**	36	40	36	36	38	38
**used PFA**	-	-	polysaccharide	polysaccharide	CERETAN MA 7008	UFC100
**d_50_ of PFA/μm**	-	-	7	7	<8	8
**content of PFA/vol.-%**	-	-	5	10	2	2
**binder content/vol.-%**	64	60	59	54	60	60

**Table 2:** Dynamic viscosity of the suspensions at different shear rates.

Zirconia Content/vol.-%	PFA: Kind and Content/vol.-%	Temperature/°C	Shear Rate/s^−1^					
			0.1	1	10	102	103	104
36	-	85	3400	312	43.80	6.86	1.43	0.52
		100	3420	309	38.30	6.03	1.07	0.32
40	-	85	4600	414	56.60	8.98	2.16	0.59
		100	3630	386	56.00	8.96	1.75	0.57
36	polysaccharide	85	3770	355	56.60	8.26	1.8	0.59
	5	100	2510	240	42.40	8.48	1.45	0.45
36	polysaccharide	85	1800	374	79.30	10.30	2.47	0.65
	10	100	5420	513	79.60	11.80	2.12	0.53
38	MA7008	85	2220	265	38.50	6.56	1.83	0.62
	2	100	1130	140	32.50	5.11	1.18	0.43
38	UFC100	85	1770	214	31.60	5.34	1.63	0.6
	2	100	1210	143	31.40	4.83	1.15	0.48
